# Genome-Wide Identification and Expression Profile Reveal Potential Roles of Peanut *ZIP* Family Genes in Zinc/Iron-Deficiency Tolerance

**DOI:** 10.3390/plants11060786

**Published:** 2022-03-16

**Authors:** Zhen Zhang, Nannan Chen, Zheng Zhang, Gangrong Shi

**Affiliations:** College of Life Sciences, Huaibei Normal University, Huaibei 235000, China; zhangzhen7550@126.com (Z.Z.); chennannan1751@126.com (N.C.); hbzhzhxyy@126.com (Z.Z.)

**Keywords:** *Arachis hypogaea*, ZRT/IRT-like protein, iron/zinc deficiency, metal uptake and transport, cultivar difference

## Abstract

Zinc/iron-regulated transporter-like protein (ZIP) family genes play crucial roles in metal uptake and transport in plants. However, little is known about their functions in peanut. Here, genome-wide analysis identified 30 peanut *AhZIP* genes that were divided into four classes. Most *AhZIP*s experienced whole-genome or segmental duplication. AhZIP proteins harbored 3–8 transmembrane domains and a typical ZIP domain, showing considerable homology with BbZIP from *Bordetella bronchiseptica*. Clustered *AhZIP*s generally share similar gene/protein structures; however, unique features were found in *AhIRT1.2*, *AhZIP1.2*, *AhZIP3.5* and *AhZIP7.8*. RNA-seq data revealed that *AhZIP2.1*/*2.2*, *AhZIP4.1*/*4.2* and *AhZIP11.1*/*11.2* were highly and preferentially expressed in roots, nodule and reproductive tissues. RT-qPCR analysis indicated that transcriptional responses of *AhZIP*s to Fe/Zn deficiency are cultivar dependent. The expressions of *AhIRT1.1*, *AhIRT1.2* and *AhZIP6.1* were closely related to Fe uptake and translocation. *AhIRT1.1* and *AhZIP7.2* expression were significantly correlated with Zn accumulation. The expression of *AhIRT1.1*, *AhIRT1.2*, *AhZIP3.6*, *AhZIP6.1* and *AhZIP11.1* was associated with Mn uptake and translocation. The results confirmed that *AhZIP* genes play crucial roles in the uptake and transport of Fe, Zn and Mn in peanut, providing clues to further functionally characterize *AhZIP* genes in the future.

## 1. Introduction

Iron (Fe) and zinc (Zn) are essential metal micronutrients for virtually all organisms. Both elements act as catalytic and structural cofactors for a large number of enzymes and play vital roles in many metabolic processes [1]. Thus, the shortage of Fe or Zn inhibits plant growth and development, posing major abiotic stresses in crop production. Most Fe and Zn in the human body are acquired from plant-based diets. However, plants are not a good source of these micronutrients because staple crops have low concentrations of Zn and Fe in edible tissues [2]. In fact, plants often suffer from Zn and Fe deficiencies due to the scarcity of Zn or low availability of Fe in the soil [3,4]. Therefore, billions of people worldwide suffer from deficiencies of these two elements, leading to nutritional disorders [5]. It is of great importance to enhance the content of Fe and Zn in edible parts of crops by improving their capacity for Fe and Zn acquirement.

The zinc/iron-regulated transporter-like protein (ZIP) family plays crucial roles in the uptake and transport of essential or nonessential divalent metals in plants, including Fe and Zn. AtIRT1, the first ZIP protein identified in *Arabidopsis thaliana*, is responsible for taking up Zn, Mn, Co, Ni and Cd from the rhizosphere to root cells [6,7,8,9]. A recent study indicates that *AtIRT1* is also specifically expressed in phloem companion cells and has a role in Fe translocation in aboveground organs [10]. AtIRT2 localizes the vesicle membrane and compartmentalizes Fe into vesicles to prevent its toxicity in the cytosol [11]. AtIRT3 is a plasma membrane localized transporter involved in the uptake of Zn and Fe in *Arabidopsis* [12]. AtZIP1 is a vacuolar transporter that is responsible for remobilizing Mn and Zn from vacuole to cytoplasm in root cells [13]. AtZIP2 is localized to the plasma membrane and may mediate Mn/Zn uptake into parenchyma cells in the xylem, contributing to xylem loading and transport of Mn/Zn to the shoot [13]. *AtZIP3* plays a role in the uptake of Zn and Fe from the soil to the plant roots, while *AtZIP4* transports Zn intracellularly or between plant tissues [14,15].

In rice (*Oryza sativa*), several *ZIP* members have been functionally characterized. OsIRT1 and OsIRT2 are Fe transporters that directly absorb the Fe^2+^, Zn^2+^, Cu^2+^, and Cd^2+^ into root cells [16,17,18]. OsZIP1 resides in the endoplasmic reticulum and plasma membrane and functions as a metal exporter in rice under Zn, Cu or Cd excess conditions [19]. OsZIP4 is a Zn transporter that may be involved in the translocation of Zn within plants [20,21]. OsZIP5 and OsZIP8 serve as the plasma membrane-localized transporter involved in Zn uptake and distribution within rice [22,23]. OsZIP7 is a plasma membrane Zn-specific transporter that plays a key role in xylem loading in roots and inter-vascular transfer in nodes to deliver Zn/Cd to developing tissues and grain in rice [24,25,26].

In contrast to the significant progress of functional characterization for individual genes, the systematic genome-wide study of *ZIP* gene family is limited. Since the first conducted by Guerinot [15], the whole genome identification of *ZIP* family has been carried out only in a few plants, including maize (*Zea mays*) [27], potato (*Solanum tuberosum*) [28], poplar (*Populus trichocarpa*) [29,30] and trifoliate orange (*Poncirus trifoliata*) [31]. Based on sequence alignments, the eukaryotic ZIP family is split into four subfamilies (ZIPI, ZIPII, gufA and LIV-1) [32]. Most of them contain 309–476 amino acid residues with eight putative transmembrane domains. The majority of ZIP proteins share a similar membrane topology where the N- and C-terminal ends are extracytoplasmic [32]. A histidine-rich domain (HRD) is contained in the long variable region of the cytoplasmic loop between TM3 and TM4, which is considered the metal binding domain playing roles in metal transport. Amphipathic TM4 and TM5 form cavities through which metal ions pass [15,32].

Peanut (*Arachis hypogaea* L., 2n = 4x = 40) is the fourth major oil crop widely grown throughout the world. It provides 20% of edible oil and 11% of food protein for global people annually. Peanut is a rich source of micronutrients, including Zn, and thus makes it more important for Zn biofortification [33]. The concentration of Zn in peanut seeds ranged from 11 to 77 mg kg^−1^, with an average of 45 mg kg^−1^ [34]. Unfortunately, peanut productivity is always affected by Fe and/or Zn deficiencies in soil because a large proportion of the crop grows in calcareous soils [33,35]. To overcome the deficiencies of Fe and Zn and to enhance their concentrations in seeds, it is necessary to fully understand the mechanism of the uptake, distribution and translocation of Fe and Zn in peanut plants.

Although several transporter genes such as *AhIRT1* [36] and *AhNramp1* [37] have been identified in peanut, the molecular mechanisms underlying the metal homeostasis remain unknown. Recently, the whole genome sequences of the cultivated peanut (*A. hypogaea* cv. Tifrunner) as well as the two wild ancestral species, *A. duranensis* and *A. ipaënsis*, have been released [38,39]. These studies make it possible for identifying gene families at the whole genome level. Here, 30 genes of the *ZIP* family were identified from cultivated peanut, and their structure, function and evolution were characterized. Moreover, the expression of *ZIP* genes in response to Fe/Zn deficiencies was evaluated. Our findings would provide clues to further characterize the functions of ZIP proteins in the uptake and translocation of Fe and Zn in peanut plants.

## 2. Results

### 2.1. Identification and Phylogenetic Analysis of the AhZIP Family in Peanut

A total of 30 putative *AhZIP* genes were identified in peanut, including four *AhIRT1*, two *AhZIP1*, two *AhZIP2*, six *AhZIP3*, two *AhZIP4*, four *AhZIP6*, eight *AhZIP7* and two *AhZIP11* (Table 1). The length of *AhZIP* genes varied from 937 bp (*AhZIP3.6*) to 8962 bp (*AhIRT1.1*), with CDS lengths from 456 bp (*AhIRT1.2*) to 1227 bp (*AhZIP4.1*). The amino acid number of AhZIP proteins ranged from 151 (AhIRT1.2) to 408 bp (AhZIP4.1), and the molecular weight varied from 16.55 kDa (AhIRT1.2) to 43.82 kDa (AhZIP4.1). The instability, GRAVY and aliphatic index of AhZIP proteins ranged from 26.98 (AhZIP2.1) to 47.10 (AhIRT1.2), from 0.167 (AhZIP1.2) to 0.767 (AhZIP11.1) and from 86.03 (AhZIP1.2) to 117.14 (AhZIP3.6), respectively. The isoelectric point (pI) of all AhZIP proteins less than 7 ranged from 5.28 (AhIRT1.2) to 6.86 (AhIRT1.3) (Table 1). TMD numbers of AhZIP proteins ranged from 3 to 8, and most of them were predicted to be plasma membrane localized except AhZIP1.2, AhZIP3.2, AhZIP3.5 and AhZIP7.8, which were predicted to localize to the endomembrane system (Table 1).

**Figure 1:**
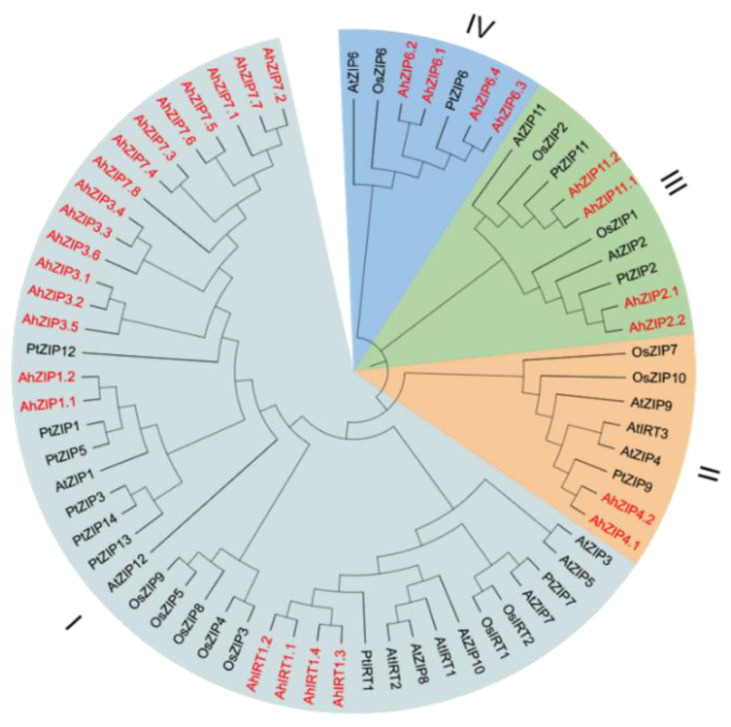
Phylogenetic relationships of ZIP proteins in peanut and other plant species. The species involved in the evolutionary tree include peanut (AhZIP), *Arabidopsis thaliana* (AtZIP), *Oryza sativa* (OsZIP), and *Poncirus trifoliata* (PtZIP). The 30 AhZIP proteins of peanut are marked in red.

To reveal the phylogenetic relationship among *AhZIP* genes, 69 ZIP protein sequences from peanut, *Arabidopsis*, rice and trifoliate orange were used to construct phylogeny with the NJ method. As reported in previous studies [29,30,31], *ZIP* members were divided into four classes: I, II, III and IV (Figure 1). Class I, the largest class, contained 20 AhZIP members. Class II consisted of AhZIP4.1 and AhZIP4.2. Class III was composed of AhZIP2.1/2.2 and AhZIP11.1/11.2. Class IV contained four orthologs of AhZIP6 (Figure 1).

### 2.2. Gene Structure, Duplication and Ka/Ks of the AhZIP Family

**Figure 2:**
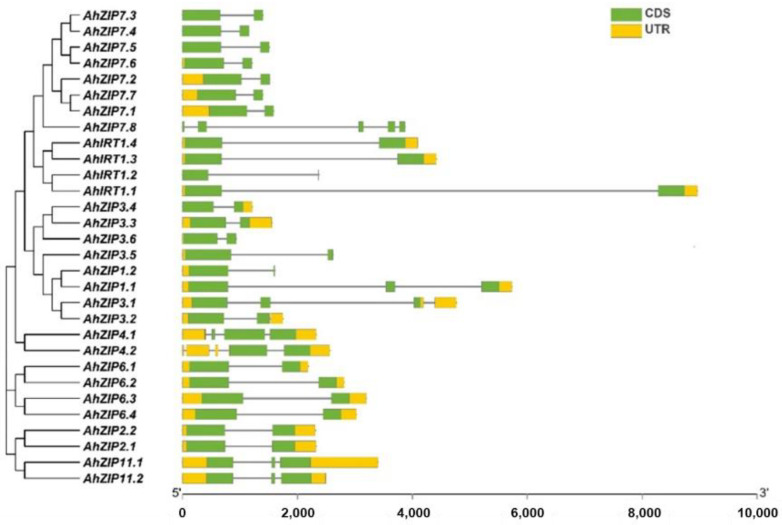
Phylogenetic relationships and exon–intron organization of *AhZIP* genes from peanut. UTR and CDS represent untranslated regions and coding sequences, respectively.

Exon–intron organizations revealed that *AhZIP* genes belonging to the same phylogenetic groups showed similar exon–intron organizations (Figure 2). Most *AhZIP* genes contained two exons with one intron. However, eight genes have distinct exon–intron structures, including *AhZIP1.1* (3 exons and 2 introns), *AhZIP3.1* (3 exons and 3 introns), *AhZIP3.2* (2 exons and 2 introns), *AhZIP4.1* (4 exons and 3 introns), *AhZIP4.2* (2 exons and 4 introns), *AhZIP11.1/11.2* (3 exons and 2 introns) and *AhZIP7.8* (5 exons and 4 introns) (Figure 2).

**Figure 3:**
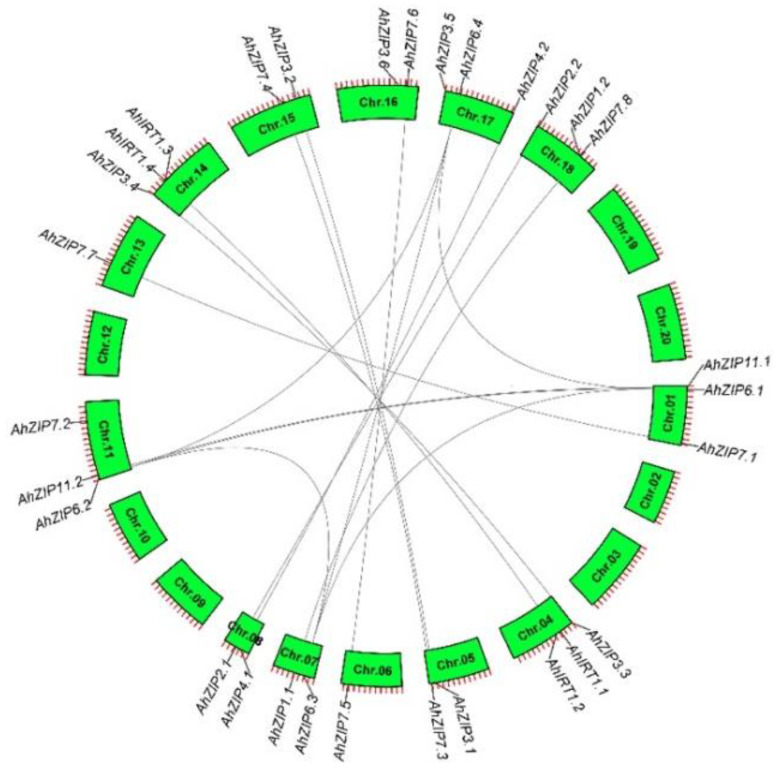
Chromosomal locations and duplications of peanut *AhZIP* genes obtained from collinearity analysis.

The 30 *AhZIP* genes were located unevenly in 13 chromosomes. A total of 13 and 17 *AhZIP* genes were identified from subgenomes A (Chr. 01–10) and B (Chr. 11–20), respectively (Figure 3). Chr. 01, 04, 11, 14, 17 and 18 contained three *AhZIP* genes, Chr. 05, 07, 08 and 15 had two genes in each chromosome, and Chr. 06 and 13 contained only one gene each, while no *AhZIP* gene was identified in Chr. 02, 03, 09, 10, 12, 19 and 20. Most of the *AhZIP* genes experienced gene duplication events except *AhIRT1.1/1.3*, *AhZIP7.2/7.8* and *AhZIP3.5/3.6*, resulting in 16 gene pairs (Figure 3). Among the duplicated genes, 12 collinear blocks resulted from whole-genome duplications (WGDs), and *AhZIP6.1/6.3* and *AhZIP6.2/6.4* resulted from segmental duplication. No tandem duplication was detected in the *AhZIP* genes. The *Ka*/*Ks* ratios (ratios of the number of nonsynonymous substitutions per nonsynonymous site/the number of synonymous substitutions per synonymous site) of all gene duplication pairs were less than 1 (Table 2), indicating that the *AhZIP* genes evolved under purifying selection [40].

### 2.3. Conserved Motifs, Domain Architectures and Models of AhZIP Proteins

**Figure 4:**
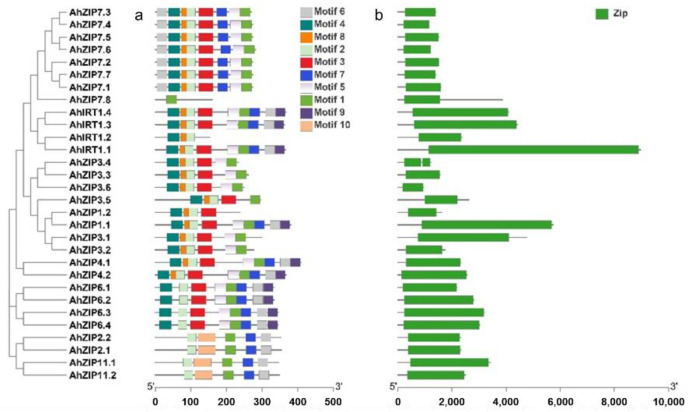
Distributions of the conserved motifs (**a**) and domains (**b**) in AhZIP proteins from peanut.

Ten conserved motifs were identified in the sequences of AhZIP proteins; among them, motifs 1, 2, 3, 5 and 9 were annotated as zinc transporters, according to the Pfam tools (Figure 4a and Table S1). All AhZIP proteins shared motifs 1 and 2 except AhZIP1.2 (without motif 1) and AhZIP7.8 (without motif 2). The distribution pattern of conserved motifs varied among phylogenetic clades, whereas it was generally similar within the same phylogenetic clades. Five proteins, including AhIRT1.2, AhZIP1.1, AhZIP1.2, AhZIP7.8 and AhZIP3.5, were found to have different motif profiles from their orthologs (Figure 4a), indicating that these proteins might have distinct functions.

All AhZIP proteins contained only one domain named ZIP, which is the typical domain of the family (Figure 4b). All AhZIP proteins were well modelled with the template, 6pgi.1.A (Figure S1 and Table S2), which is the A chain of the BbZIP protein from *Bordetella bronchiseptica*. BbZIP has been revealed to have a binuclear metal center, where two metal ions were trapped halfway through the membrane and the two metal-binding sites play asymmetric roles within the transport pathway [41]. Sequence identity ranged from 13.27% to 20.16%, the value of GMQE ranged from 0.12 to 0.54 and QMEANDisCo global score ranged from 0.32 to 0.54 (Table S2). These data are suggestive of the high quality of the 3D model predictions on AhZIP proteins.

**Figure 5:**
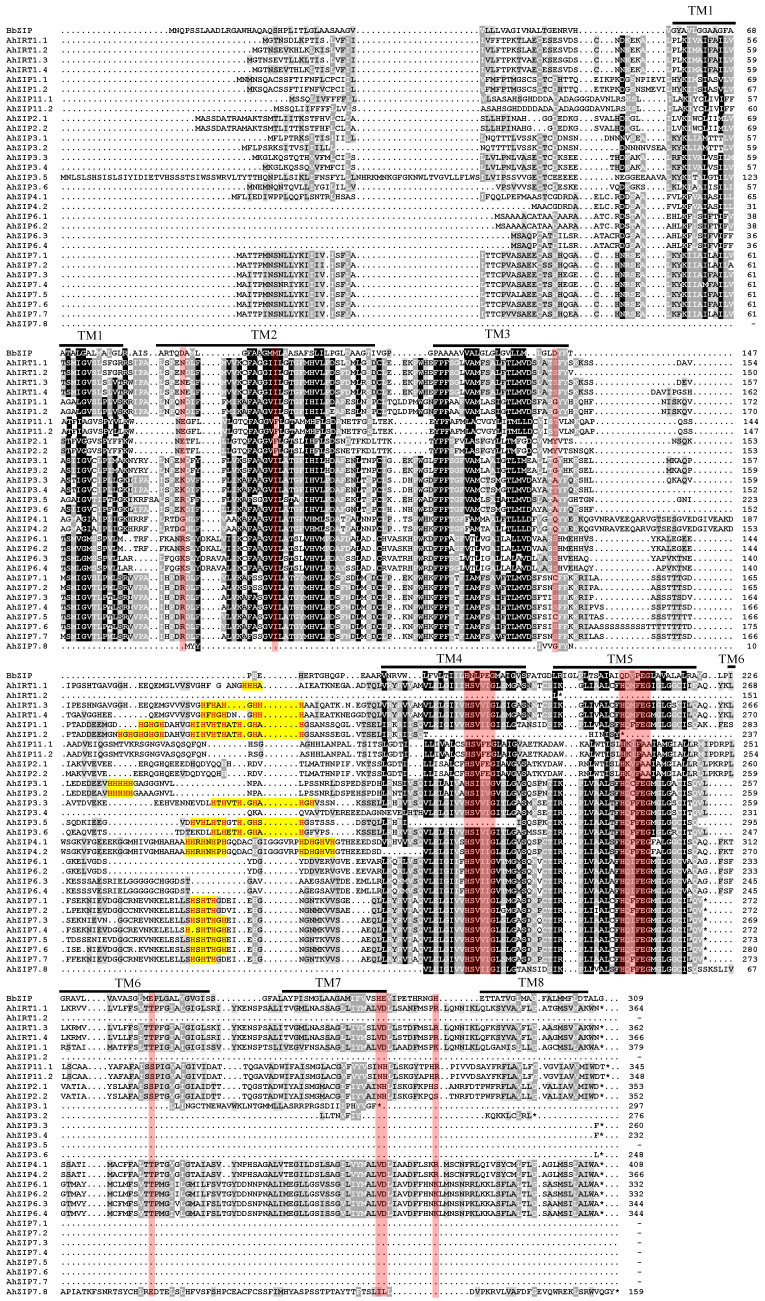
Multiple sequence alignment of BbZIP and AhZIP proteins. BbZIP protein from *Bordetella bronchiseptica* and 30 AhZIP proteins were aligned using ClustalW. The red bars indicate that amino acid residues possibly formed Zn^2+^ and Cd^2+^ binding sites in the BbZIP protein. The yellow highlighted motifs indicate histidine-rich domains (HRDs) in the long cytoplasmic loop between TM3 and TM4.

Multiple sequence alignment showed considerable homology between the BbZIP and AhZIP proteins throughout the TMD, particularly in TM2, TM4 and TM5 (Figure 5). The TMD structure of AhZIP proteins showed great diversity. Among the 30 AhZIP proteins, only 14 have the 3 + 5 TMD structure (Figure 5). AhIRT1.2, AhZIP1.2 and AhZIP3.5 only contained the first three TMDs (TM1–TM3), AhZIP7.8 contained the last four TMDs (TM5–TM8), and the remaining homologs of AhZIP3 and AhZIP7 contained the first five TMDs (TM1–TM5) (Figure 5). A long chain variable region was found between TM3 and TM4 in most AhZIPs, except for AhIRT1.2 and AhZIP7.8, and most of them contain various HRDs such as HXHXH, HHH, HHHHH, HXHXHXH and HHXHXHXH (Figure 5). Additionally, glycine (G) residues are always found near or inside HRDs (Figure 5).

### 2.4. Expression Profiles of AhZIP Genes in Different Tissues of Peanut

**Figure 6:**
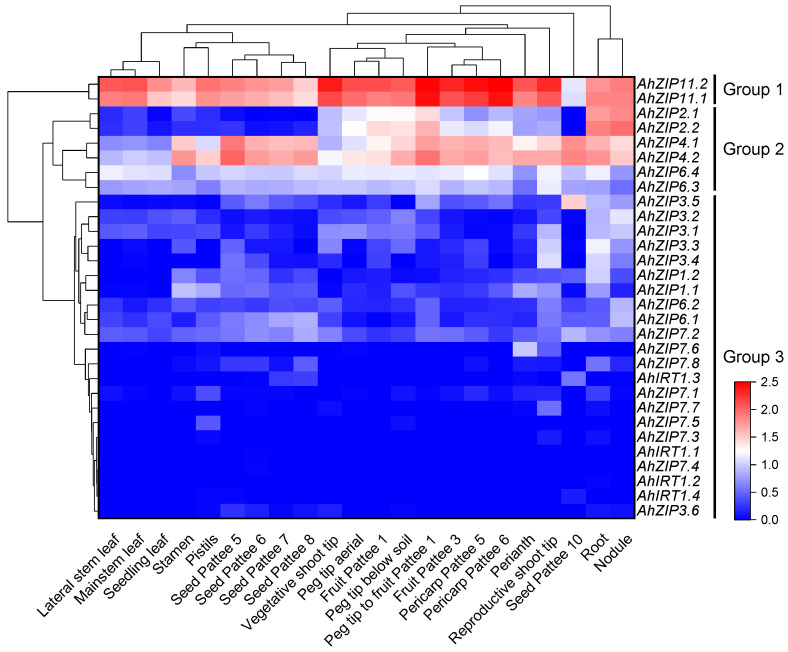
Expression profiles of *AhZIP* genes across the different tissues. Gene expression is expressed in lg(TPM + 1). Pattee 1, 3, 5, 6, 7, 8 and 10 represent different developmental stages of peanut pods according to Pattee et al. [42], who classified peanut pod maturity into 15 categories.

The RNA-seq data showed that all *AhZIP* genes were tissue-specifically expressed in peanut plants (Table S3). Generally, the gene expression profiles were similar within the same phylogenetic classes. The 30 *AhZIP* genes could be classified into three distinct groups according to the gene expression patterns (Figure 6). Group 1 consists of two orthologs of *ZIP11* that showed the highest gene expression levels in almost all tissues tested. Group 2 contained *AhZIP4.1/4.2*, *AhZIP6.3/6.4* and *AhZIP2.1/2.2*, representing intermediate levels of gene expression. Group 3 is composed of the remaining 22 genes; these genes were not express in most peanut tissues or showed low expression levels. *AhZIP2.1/2.2* is specifically and highly expressed in the root and nodule. By contrast, *AhZIP4.1/4.2* was mainly expressed in roots, nodules and reproductive tissues (i.e., peg tip to fruit, seed and pericarp); *AhZIP11.1/11.2* was expressed in all tissues but was relatively higher in reproductive tissues (i.e., peg tip to fruit, fruit and pericarp) and vegetative shoot tips (Figure 6).

### 2.5. Gene Expression of AhZIPs in Response to Fe- and Zn-Deficiency

**Figure 7:**
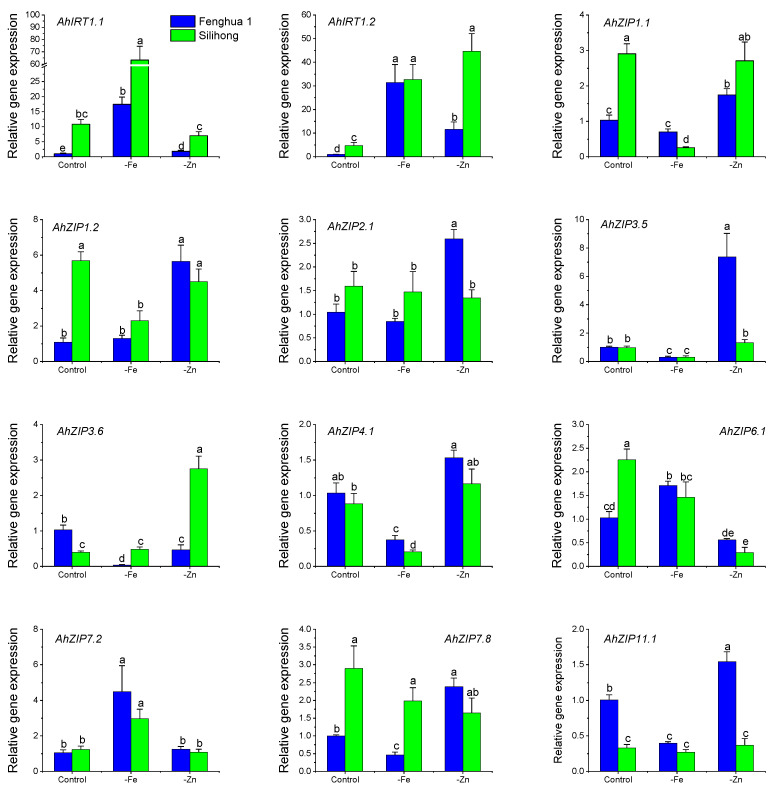
Expression levels of 12 *AhZIP* genes in the root of two peanut cultivars in response to Fe/Zn deficiency. Data (means ± SE, *n* = 4) sharing the same letter(s) above the error bars are not significantly different at the 0.05 level based on the Duncan multiple range test.

The expression of *AhZIP* genes differed between the two cultivars in response to Fe- and Zn-deficiency (Figure 7). Under the control condition, Fenghua 1 showed higher expressions of *AhZIP3.6* and *AhZIP11.1* than Silihong, while Silihong showed higher expressions of *AhIRT1.1*, *AhIRT1.2*, *AhZIP1.1*, *AhZIP1.2*, *AhZIP6.1* and *AhZIP7.8* than Fenghua 1. Under Fe-deficiency condition, Fenghua 1 showed higher expressions of *AhZIP1.1* and *AhZIP4.1* than Silihong, while Silihong showed higher expressions of *AhIRT1.1*, *AhZIP3.6* and *AhZIP7.8* than Fenghua 1 (Figure 7). Under Zn-deficiency condition, Fenghua 1 showed higher expressions of *AhZIP2.1*, *AhZIP3.5* and *AhZIP11.1* than Silihong, while Silihong showed higher expressions of *AhIRT1.1*, *AhIRT1.2* and *AhZIP3.6* than Fenghua 1 (Figure 7).

All *AhZIP* genes tested transcriptionally responded to Fe- and Zn-deficiency in peanut roots dependent on cultivars (Figure 7). Fe-deficiency enhanced the expressions of *AhIRT1.1*, *AhIRT1.2* and *AhZIP7.2* but reduced those of *AhZIP3.5* and *AhZIP4.1* for both cultivars, while other genes showed cultivar differences in response to Fe-deficiency. Fe-deficiency inhibited the expressions of *AhZIP1.1*, *AhZIP1.2* and *AhZIP6.1* in Silihong, while they were not affected or increased in Fenghua 1. The expressions of *AhZIP3.6*, *AhZIP7.8* and *AhZIP11.1* in Fenghua 1 were inhibited by Fe-deficiency, while in Silihong, they were unaffected.

Zn-deficiency upregulated the expression of *AhIRT1.2* for both cultivars, while other genes showed cultivar differences in response to Zn-deficiency (Figure 7). The influence of Zn-deficiency on gene expression was more pronounced in Fenghua 1 than in Silihong. The expressions of *AhIRT1.1*, *AhZIP1.1*, *AhZIP1.2*, *AhZIP2.1*, *AhZIP3.5*, *AhZIP7.8* and *AhZIP11.1* were induced by Zn-deficiency in Fenghua 1, while that in Silihong were unchanged or downregulated. In contrast, *AhZIP3.6* were significantly induced by Zn-deficiency in Silihong, whereas in Fenghua 1, it was downregulated.

### 2.6. Metal Accumulation and Translocation in Response to Fe- or Zn-Deficiency

**Figure 8:**
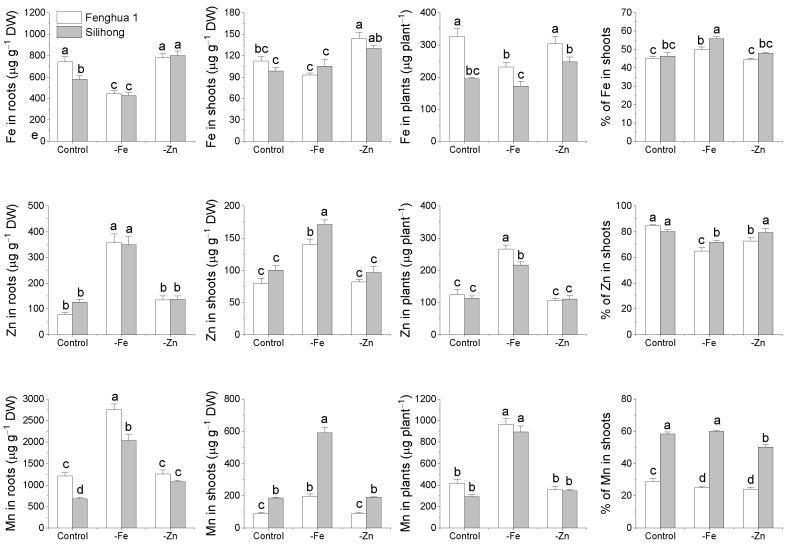
The accumulation and translocation of Fe, Mn and Zn in two peanut cultivars in response to Fe- or Zn-deficiency. Data (means ± SE, *n* = 4) shared the same letter(s) above the error bars are not significantly different at the 0.05 level based on the Duncan multiple range test.

The two peanut cultivars differed from each other in the accumulation of Fe and Mn (Figure 8). The concentration of Fe in roots and shoots as well as the total amount of Fe in plants were significantly higher in Fenghua 1 than in Silihong. Compared with Silihong, Fenghua 1 showed higher root Mn concentrations but lower shoot Mn concentrations, which resulted in a lower root-to-shoot Mn translocation (Figure 8). Fe-deficiency significantly reduced Fe concentrations in roots for both cultivars; however, shoot Fe concentrations were unchanged. This contributed to an increase in Fe translocation in Fe-deficient plants. The total amount of Fe in plants was decreased by Fe-deficiency in Fenghua 1 but was unaffected in Silihong. Zn-deficiency increased shoot Fe concentrations for both cultivars, while root Fe concentrations, total Fe in plants and the percentage of Fe in shoots were not affected.

The concentrations of Zn in roots and shoots as well as the total amount of Zn in plants were significantly enhanced by Fe-deficiency for both cultivars, while the percentage of Zn in shoots was decreased. Zn-deficiency did not change Zn accumulation, but reduced Zn translocation from roots to shoots in Fenghua 1 (Figure 8). Fe-deficiency significantly increased Mn concentrations in roots and shoots as well as the total amount of Mn in plants for both cultivars. The percentage of Mn in shoots declined by Fe-deficiency in Fenghua 1, while it was unaffected in Silihong. Zn-deficiency reduced Mn translocation for both cultivars, while root Mn concentrations in Silihong were increased (Figure 8).

### 2.7. Relationship of Gene Expression of AhZIPs and Metal Accumulation

To identify the *AhZIP* genes involved in metal uptake and translocation in peanut roots, a stepwise linear regression analysis was performed. As showed in Table 3, the expression of *AhIRT1.1* is significantly correlated with the total amount of Fe in plants, concentrations of Zn and Mn in shoots, and the percentage of Fe and Mn in shoots. The expression of *AhIRT1.2* is significantly correlated with Fe concentrations in roots, the total amount of Mn in plants, and the percentage of Mn in shoots. The expression of *AhZIP3.6* is significantly correlated with Mn concentrations in shoots and the percentage of Mn in shoots. The expression of *AhZIP6.1* is significantly correlated with Fe concentrations in roots and shoots. The expression of *AhZIP7.2* is significantly correlated with Zn concentrations in roots and the total amount of Zn in plants. The expression of *AhZIP11.1* is significantly correlated with Mn concentrations in shoots.

## 3. Discussion

In this study, 30 *ZIP* members were identified in peanut, which is the largest compared with the reported plant species. For example, 12 *ZIP* genes were identified in most plant species such as potato, trifoliate orange and maize, while 15, 16 and 21 *ZIP* members were reported in *Arabidopsis*, rice and *P. trichocarpa*, respectively [27,28,29,30,31]. Peanut is an allotetraploid species that essentially contains two complete sets of subgenome (A and B) from two diploid ancestral species: *A. duranensis* (AA) and *A. ipaensis* (BB) [39]. Here, we showed that all *AhZIP*s are multicopy genes, and most of them resulted from WGDs (Figure 3). *AhZIP* genes are unevenly distributed in the two subgenomes of peanut. The subgenome A contained 13 *AhZIP* genes, while 17 genes were distributed in the subgenome B (Figure 3). This phenomenon indicates that gene loss or gain occurred during the evolutionary process.

In agreement with previous studies [27,30,31], most of the AhZIP proteins were predicted to localize to the plasma membrane, and AhZIP1.2, AhZIP3.2, AhZIP3.5 and AhZIP7.8 localize to the endomembrane system (Table 1). AtIRT1, AtIRT3 and AtZIP2 from *Arabidopsis* have been confirmed to be localized to the plasma membrane [9,12,13], while AtIRT2 and AtZIP1 localized to the vesicle or vacuolar membranes [11,13]. In rice, OsIRT1, OsIRT2, OsZIP1, OsZIP4, OsZIP5, OsZIP7 and OsZIP8 have been proven to localize to the plasma membrane [16,17,19,20,21,22,23,24,25,26].

AhZIPs showed a wide variation in TMDs, ranging from 3 to 8 TMDs (Table 1). More than half of AhZIPs have 3–5 TMDs, which is not consistent with the results of Guerinot [15], who proposed that ZIP proteins typically contained 8 TMDs. The variation in TMD number in ZIP proteins has been reported in several plant species such as maize (6–13 TMDs), potato (6–9 TMDs), trifoliate orange (6–9 TMDs) and *P. trichocarpa* (3–13 TMDs) [27,28,30,31]. The ZIP family was generally predicted to have a 3 + 5 TMD architecture [15]. However, the TMD structure is more diverse in AhZIP proteins. Among 30 AhZIP proteins, only 14 have the 3 + 5 TMD structure (Figure 5). The unusual arrangement of TMDs suggests that the peanut AhZIP family has a distinctive evolutionary process and significant divergence of physiological functions.

The HRD within the long cytoplasmic loop between TM3 and TM4 has been postulated to serve as a potential Zn^2+^ binding site in many ZIPs [15,43]. The histidine residues have been proven to coordinate Zn^2+^ in the intracellular loop between TM3 and TM4 of human hZIP4 [43]. In the current study, all AhZIP proteins except AhIRT1.2 and AhZIP7.8 have the large intracellular loop and most of them contain HRDs including HXHXH, HHH, HHHHH, HXHXHXH and HHXHXHXH (Figure 5). Additionally, a glycine (G) residue is always found near or inside HRDs in the intracellular loop between TM3 and TM4 of AhZIP proteins. Moreover, almost all TMDs contained a conserved glycine (Figure 5). These findings indicate that glycine residues might be essential for the structure and function of AhZIP proteins. In human hZIP4, six conserved G residues (G330, G374, G512, G526, G535 and G630) were identified to be located at or near the TM–TM interface, suggesting a role in mediating TM packing [41].

All AhZIP proteins contained the typical domain of the family, zip, and are perfectly modeled on the template, 6pgi.1.A (the A chain of BbZIP protein from *B. bronchiseptica*). BbZIP as a Zn^2+^ and Cd^2+^ transporter has a binuclear metal center, where two metal ions are trapped halfway through the membrane, and the two metal-binding sites play asymmetric roles within the transport pathway [41]. A pairwise sequence alignment showed considerable homology between BbZIP and AhZIPs throughout the TMD (Figure 5). BbZIP has been proven to have six Zn^2+^ binding sites and one Cd^2+^ binding site, which were formed by 13 amino acid residues including D89 from TM2; M99 and D144 from TM3; H177, N178 and E181 from TM4; Q207, D208 and E211 from TM5; E240 from TM6; H275 and E276 from TM7; and H286 from TM8 [41]. Almost all of them were mapped to conserved amino acid residues of AhZIP proteins, particularly the two metal-binding motifs “^177^HNLPEG^182^” from TM4 and “^207^QDVPEG^212^” from TM5, which form the binuclear metal center of BbZIP: M1 (H177, E181, Q207 and E211) and M2 (N178, E181, D208, E211, and E239 from TM6) (Figure 5). The similar structures suggest that AhZIPs might have equivalent physiological functions as BbZIP.

The 30 AhZIP members were divided into four classes: I, II, III and IV (Figure 1). The classification concurred with those reported in previous studies [29,30,31]. Class I, as the largest class, contained 20 AhZIP members, including eight orthologs of AhZIP7, six orthologs of AhZIP3, four orthologs of AhIRT1 and two orthologs of AhZIP1. Most of the AhZIPs belonging to class I shared six motifs (4, 8, 2, 3, 5 and 1), except for AhZIP7.8, AhIRT1.2, AhZIP3.5 and AhZIP1.2, which have less motifs (Figure 4a). Motifs 1, 2, 3 and 5 were annotated as zinc transporters (Table S1). The motif composition indicates that the class I proteins might be responsible for metal transport in peanut.

It is noteworthy that several genes belonging to class I significantly differed from other orthologous genes in the gene/protein structures. AhZIP7.8, encoding 159 aa with four TMDs (TM5-TM8) and one motif (motif 1), has a distinctive gene structure (five exons and four introns). AhIRT1.2 contained three motifs and three TMDs, which are greatly less than the other three orthologs. AhZIP1.2 has three TMDs and four motifs, which are less than half of AhZIP1.1. *AhZIP1.2* contained two exons and one intron, while *AhZIP1.1* contained three exons and two introns. AhZIP3.5 contained three TMDs (TM1–TM3) and five motifs, while other orthologs contained five TMDs (TM1–TM5) and six motifs. Moreover, AhZIP7.8, AhZIP3.5 and AhZIP1.2 were predicted to be located in the endomembrane system. The distinctive features indicate that these genes might differ from their orthologous genes in physiological functions.

Class II consisted of AhZIP4.1 and AhZIP4.2. The two members are significantly similar in protein structure and physiochemical traits, implying similar functions in peanut plants. However, they differed from each other in gene structure. *AhZIP4.1* contained four exons and three introns, while *AhZIP4.2* contained two exons and four introns. The difference in exon/intron organization indicates a significant gene divergence during the evolutionary process. Class III includes *AhZIP2.1*/*2.2* and *AhZIP11.1*/*11.2*. The four members shared a similar motif composition (motifs 1, 2, 6, 7 and 10). However, the gene structure is different between *AhZIP2* (two exons and one intron) and *AhZIP11* (three exons and two introns). Class IV contained four orthologous genes of *AhZIP6*, which were derived from both WGD and segmental duplication. The four members are greatly similar in physiochemical features, TMDs, subcellular location, and gene/protein structures, indicating similar physiological functions. The *Ks* values of *AhZIP6.1/6.3* (1.3068) and *AhZIP6.2/6.4* (1.3548) were considerably higher than that of *AhZIP6.1/6.2* (0.0477) and *AhZIP6.3/6.4* (0.0485) (Table 2), indicating that segmental duplication may occur earlier than WGD.

Gene duplication is a major source of novel genes, contributing to the evolution of new functions [44]. However, before the functional divergence, duplicated genes are usually functionally redundant, which may induce gene loss [45]. To avoid gene loss during evolution processes, the expression of duplicated genes is reduced compared with the ancestral gene [45]. In the present study, 22 *AhZIP* genes, including all members of class I as well as two orthologs of *AhZIP6* (*AhZIP6.1*/*6.2*), showed low expression in the 22 peanut tissues under normal conditions, and most of them have more than four orthologous genes (Figure 6). Our results are in accordance with those of Qian et al. [45], suggesting that the low expression of these genes might be beneficial for long-term maintenance of duplicate genes and their functional redundancy. Despite this, the RT-qPCR results indicated that these low expressed genes in peanut roots can be induced by Fe- and Zn-deficiency in a cultivar-dependent manner (Figure 7). It was observed that *AhZIP2.1/2.2*, *AhZIP4.1/4.2* and *AhZIP11.1/11.2* were preferentially expressed in roots, nodule and reproductive tissues (i.e., peg tip to fruit, seed and pericarp) (Figure 6). These genes might be involved in metal uptake and translocation by the root and might be responsible for the development of pods or seeds by regulating the metal transport.

The expression of *AhZIP* genes showed wide differences in response to Fe- and Zn-deficiency depending on cultivars (Figure 7). Fe-deficiency enhanced the expressions of *AhIRT1.1* and *AhIRT1.2* for both cultivars. Moreover, the expression of *AhIRT1.2* was also upregulated by Zn-deficiency for both cultivars. Phylogenetic analysis showed that AhIRT1.1 and AhIRT1.2 have a close relationship with AtIRT1 and AtIRT2 (Figure 1). AtIRT1 is a Fe transporter responsible for taking up Zn, Mn, Co, Ni and Cd from the rhizosphere to root cells [6,7,8,9]. AtIRT2 is involved in the compartmentalization of Fe into vesicles to avoid its toxicity in the cytosol [11]. Fe-deficiency significantly increased the concentration of Zn and Mn in roots and shoots as well as the total amount of Zn and Mn in plants for both cultivars (Figure 8). The expression of *AhIRT1.1* significantly correlated with the total amount of Fe in plants, concentrations of Zn and Mn in shoots, and the percentage of Fe and Mn in shoots (Table 3). The expression of *AhIRT1.2* significantly correlated with Fe concentrations in roots, the total amount of Mn in plants and the percentage of Mn in shoots (Table 3). These findings suggested that *AhIRT1.1* and *AhIRT1.2* might be responsible for the uptake of Fe, Zn and Mn in peanut plants. *PtIRT1* from trifoliate orange, which was closely related to *AhIRT1.1* and *AhIRT1.2*, has been proven to be responsible for Fe, Zn and Mn uptake [31].

Fe-deficiency induced the expression of *AhZIP7.2* for both cultivars. The stepwise linear regression analysis revealed that the expression of *AhZIP7.2* significantly correlated with Zn concentrations in roots and the total amount of Zn in plants (Table 3). The results indicate that *AhZIP7.2* might be involved in Fe/Zn uptake in peanut plants. The expression of *AhZIP3.6* was positively correlated with Mn concentrations in shoots and the percentage of Mn in shoots (Table 3), indicating a possible role in Mn translocation from roots to shoots. Phylogenetic analysis showed that AhZIP1/3/7 has a closer relationship with OsZIP3/4/5/8/9 (Figure 1). OsZIP3 is most likely to be localized at the plasma membrane and responsible for xylem unloading of Zn in the nodes of rice [46]. OsZIP4 is a Zn transporter involved in the translocation of Zn within plants [20,21]. OsZIP5 and OsZIP8 serve as the plasma membrane-localized transporter involved in Zn uptake and distribution within rice [22,23]. *OsZIP5* and *OsZIP9* are tandem duplicates that act synergistically in Zn/Cd uptake [47].

The expression of *AhZIP6.1* was repressed by Zn-deficiency, while its response to Fe-deficiency was dependent on cultivars (Figure 7). Fe-deficiency upregulated the expression of *AhZIP6.1* in Fenghua 1 but downregulated that in Silihong. The expression of *AhZIP6.1* is significantly correlated with Fe concentrations in roots and shoots (Table 3). These data indicate that *AhZIP6.1* might be responsible for the transport of Fe in peanut plants. Phylogenetic analysis showed that *AhZIP6.1* has a close relationship to *OsZIP6* (Figure 1), which is suggested to competitively take up Fe^2+^ and Co^2+^ [48]. TcZNT6 of *Thlaspi caerulescens* is a metal transporter that is responsible for the transport of Zn, Cd, and Fe or Mn [49]. ZIP6 from *Arabidopsis halleri* mainly expressed in vascular tissues and encodes a Zn/Cd transporter [50].

The expression of *AhZIP11.1* in Fenghua 1 was inhibited by iron deficiency but induced by zinc deficiency, whereas its expression in Silihong was not affected (Figure 7). Stepwise linear regression analysis revealed that the expression of *AhZIP11.1* was significantly correlated with Mn concentrations in shoots (Table 3). It seems that *AhZIP11.1* is involved in Mn transport in peanut. Similarly, *PtZIP11* from trifoliate orange has been suggested to be involved in Mn transport but not Zn or Fe [31]. However, *AtZIP11* from *Arabidopsis* [13] and *NtZIP11* from *Nicotiana tabacum* [51] were shown to mediate Zn uptake but not Fe or Mn.

In agreement with previous studies [52], we found that Silihong showed a higher capacity for tolerance of Fe-deficiency than Fenghua 1 (Figure 8). This might be attributed to lower Fe requirement under normal conditions and higher Fe translocation in case of iron deficiency. In addition, the higher expression of *AhIRT1.1* in the root of Silihong under iron deficiency might enhance its capacity for Fe acquirement, therefore contributing to tolerance of Fe-deficiency. Moreover, Silihong has a higher capacity for the translocation of Mn from roots to shoots than Fenghua 1 (Figure 8). Stepwise linear regression analysis revealed that the expressions of *AhIRT1.1*, *AhIRT1.2*, and *AhZIP3.6* were significantly related to the translocation of Mn in peanut plants (Table 3). Silihong showed higher expressions of *AhIRT1.1* and *AhZIP3.6* than Fenghua 1. Thus, a higher capacity for Mn translocation in Silihong might result from the higher expression of *AhIRT1.1* and *AhZIP3.6*.

## 4. Materials and Methods

### 4.1. Identification of ZIP Genes in Peanut

The protein sequences of 15 AtZIPs from *Arabidopsis* and 12 OsZIPs from rice were retrieved from Phytozome database (https://phytozome-next.jgi.doe.gov/, accessed on 12 August 2021). The sequences obtained were used as queries for BLASTP against the genome of cultivated peanut (cv. Tifrunner) on Phytozome. All candidates were further examined with the hmmscan tool (https://www.ebi.ac.uk/Tools/hmmer/search/hmmscan, accessed on 15 August 2021). The sequences that contain the ZIP domain (Pfam: PF02535) were recognized as AhZIP proteins. Thereafter, physiochemical parameters including molecular weight, amino acid number, GRAVY and pI were analyzed using the ProtParam tool (https://web.expasy.org/protparam/, accessed on 27 August 2021) [53]. The TMDs of AhZIPs were identified using TOPCONS (http://topcons.net/, accessed on 1 September 2021) [54]. Subcellular targeting sites of AhZIP proteins were predicted using Plant-mPLoc (http://www.csbio.sjtu.edu.cn/bioinf/plant-multi/, accessed on 31 August 2021) [55].

### 4.2. Phylogenetic and Structural Analysis of AhZIP Proteins

Sequences of ZIP proteins of *Arabidopsis*, rice and trifoliate orange retrieved from Phytozome database were aligned using ClustalW integrated in MEGA-X software (v. 10.2.6). Based on the sequence alignment, a phylogenetic tree was constructed by the neighbor-joining (NJ) method with the No. of no-difference models. A bootstrap test of 1000 replicates was used for estimating the reliability of the phylogenetic tree. The tree was drawn and modified using iTOL (https://itol.embl.de/itol.cgi, accessed on 5 September 2021) [56].

The conserved motifs and domains of AhZIP proteins were analyzed using the MEME (https://meme-suite.org/meme/tools/meme, accessed on 31 January 2022) [57] and Pfam tool (http://pfam.xfam.org/search#tabview=tab1, accessed on 2 February 2022) [58], respectively, and were visualized using TBtools software [59]. Their homology-modelled 3D structures were predicted using the SwissModel (https://swissmodel.expasy.org/, accessed on 5 February 2022) [60].

### 4.3. Structure, Duplication and Ka/Ks of AhZIP Genes

The exon/intron structures of *AhZIP* genes were detected with genomic and coding sequences using GSDS v. 2.0 (http://gsds.gao-lab.org/, accessed on 11 September 2021) [61]. Gene collinearity and *Ka*/*Ks* were analyzed by the One Step MCScanX and Simple *Ka*/*Ks* calculator (NJ) integrated in the TBtools software, respectively [59]. Diagrams of the exon/intron organization and gene duplication event were drawn using the TBtools software [59].

### 4.4. Gene Expression Analysis Based on RNA-Seq Data

The expression profiles of the *AhZIP* genes from Tifrunner were identified using RNA-seq data obtained from the PeanutBase database (https://www.peanutbase.org/, accessed on 6 February 2022) [62]. The read counts were transformed to TPM (Transcripts Per Kilobase of exon model per Million mapped reads), and the heatmap diagram was constructed with lg(TPM + 1) using TBtools [59].

### 4.5. Plant Growth, Metal Determination and RT-qPCR Analysis

Two peanut cultivars differing in Fe-deficiency tolerance, Fenghua 1 (Fe-deficiency sensitive cultivar) and Silihong (Fe-deficiency tolerant cultivar), were used for determining the relationships between the expression of *AhZIP* genes and metal accumulation in peanut plants [52]. After surface sterilized with 5% sodium hypochlorite (1 min), the seeds were presoaked in distilled water for 24 h, and then, they were sown in sand for germination. Three-day-old uniform seedlings were transferred to polyethylene pots and cultured as previously reported [63]. The seven-day-old seedlings were treated with Fe- (without Fe) or Zn-deficiency (without Zn) in hydroponic cultures, with the normal nutrition solution containing 50 μM Fe-EDTA and 3.8 μM ZnSO_4_ as the control [64]. The experiment was arranged in a randomized complete design with four replications (pots) for each treatment. Each replication includes three seedlings. During the growing period, pots were randomly arranged and moved daily for minimizing position effects. After 14 days of treatment, plants were harvested and fresh tissues of roots were sampled for RT-qPCR analysis.

The harvested plants were separated into roots and shoots and oven-dried to constant weight. After weighing, plant tissues were ground into powder and were digested with a mixture of HNO_3_ and HClO_4_ (3:1, *v*/*v*) using the method described by Su et al. [63]. The levels of Fe, Zn and Mn were determined by flame atomic absorbance spectrometry (WFX-110, Beijing Rayleigh Analytical Instrument Company, Beijing, China).

The expression levels of 12 *AhZIP* genes representing unique gene and protein structures were detected using RT-qPCR with the method described previously [52] and with *Ah60S* as the endogenous reference gene. The primers are listed in Table S4. Four biological replicates were performed for each treatment, and three technical replicates were performed for each sample. The relative gene expression was calculated using the 2^−ΔΔCT^ method [65].

### 4.6. Statistical Analysis

Data were subjected to one-way ANOVA, and significant variations among means were determined by the Duncan’s multiple-range test at 0.05 probability. Stepwise linear regression analysis was performed on the expression of *AhZIP* genes and metal accumulation. All statistical analyses were conducted using IBM SPSS Statistics v. 22 (IBM, New York, NY, USA).

## 5. Conclusions

In conclusion, a total of 30 *ZIP* genes were identified in peanut, which were divided into four classes. All AhZIP proteins contained the typical zip domain and are perfectly modeled on the 6pgi.1.A template, suggesting a role of metal transport in peanut. Unlike previous reports, AhZIP proteins showed a wide variation in TMDs (3–8 TMDs) and only 14 AhZIPs have the 3 + 5 TMD structure. Clustered *AhZIP*s generally share similar gene/protein structures; however, unique features were found in *AhIRT1.2*, *AhZIP1.2*, *AhZIP3.5* and *AhZIP7.8*. Most *AhZIP* genes showed reduced expression under normal conditions, while *AhZIP2.1*/*2.2*, *AhZIP4.1*/*4.2* and *AhZIP11.1*/*11.2* are highly and preferentially expressed in roots, nodule and reproductive tissues, suggesting an essential role in pod and seed development. Transcriptional responses of *AhZIP*s to Fe/Zn deficiency in peanut roots are dependent on cultivar, which might be, at least partially, responsible for the different metal accumulation between Fenghua 1 and Silihong. The findings provide essential information to further functionally characterize *AhZIP* genes in the uptake and translocation of metal ions in peanut plants, which is great of importance for screening or breeding cultivars for Fe/Zn biofortification.

## Figures and Tables

**Table 1:** Molecular characterization of *AhZIP* genes identified in peanut.

Gene Name	Gene ID	Gene Length (bp)	CDS (bp)	MW ^a^ (kDa)	Aa ^b^	Instability	Aliphatic Index	GRAVY ^c^	pI ^d^	No. of TMD ^e^	Location
*AhIRT1.1*	arahy.T4CX6H	8962	1095	38.94	364	33.45	109.29	0.55	6.3	8/out-out	PM ^f^
*AhIRT1.2*	arahy.B3ZT22	2373	456	16.55	151	47.1	109.07	0.654	5.28	3/out-in	PM
*AhIRT1.3*	arahy.234RNS	4415	1089	39.25	362	36.8	111.99	0.573	6.86	8/out-out	PM
*AhIRT1.4*	arahy.8VMZ7D	4094	1101	39.56	366	37.5	108.63	0.53	6.34	8/out-out	PM
*AhZIP1.1*	arahy.XJH13Y	5732	1140	40.51	379	41.77	104.01	0.477	6.29	8/out-out	PM
*AhZIP1.2*	arahy.ZLZ7ZM	1608	714	25.74	237	44.01	86.03	0.167	6.53	3/out-in	EMS ^g^
*AhZIP2.1*	arahy.VX1J70	2322	1062	38.77	353	26.98	107.48	0.495	6.52	8/in-out	PM
*AhZIP2.2*	arahy.1Q0IUD	2309	1059	38.71	352	27.91	103.35	0.472	6.62	8/in-out	PM
*AhZIP3.1*	arahy.CK2LDM	4766	894	32.38	297	39.12	107.71	0.254	6.03	5/out-in	PM
*AhZIP3.2*	arahy.E7VKLQ	1747	831	30.12	276	38.03	110.62	0.288	6.62	5/out-in	EMS
*AhZIP3.3*	arahy.ZVRF07	1554	783	28.26	260	33.11	107.19	0.423	6.08	5/out-in	PM
*AhZIP3.4*	arahy.30BR38	1211	699	25.09	232	33.22	109.66	0.568	5.28	5/out-in	PM
*AhZIP3.5*	arahy.WQ3KQR	2618	888	32.26	295	37.42	99.49	0.212	6.45	3/out-in	EMS
*AhZIP3.6*	arahy.05ZQZB	937	747	26.99	248	31.92	117.14	0.524	5.91	5/out-in	PM
*AhZIP4.1*	arahy.KIJ6L7	2325	1227	43.82	408	42.93	95.22	0.324	6.02	8/out-out	PM
*AhZIP4.2*	arahy.2FF2JF	2561	1101	38.83	366	41.96	95.25	0.34	6.02	8/out-out	PM
*AhZIP6.1*	arahy.58GIJL	2185	999	35.60	332	35.92	106.99	0.631	5.77	8/out-out	PM
*AhZIP6.2*	arahy.78T540	2812	999	35.54	332	35.89	107.29	0.647	5.9	8/out-out	PM
*AhZIP6.3*	arahy.0DI5A2	3196	1035	36.29	344	37.88	106.1	0.629	6.29	8/out-out	PM
*AhZIP6.4*	arahy.E6QUMC	3026	1035	36.31	344	37.66	106.37	0.630	6.29	8/out-out	PM
*AhZIP7.1*	arahy.BTX8K3	1585	819	29.67	272	45.6	103.2	0.400	6.10	5/out-in	PM
*AhZIP7.2*	arahy.092K8V	1514	819	29.61	272	44.17	104.3	0.413	6.58	5/out-in	PM
*AhZIP7.3*	arahy.RP74H9	1398	810	29.45	269	45.52	109.44	0.412	6.04	5/out-in	PM
*AhZIP7.4*	arahy.FX0GN0	1151	819	29.88	272	44.84	103.57	0.347	6.62	5/out-in	PM
*AhZIP7.5*	arahy.1C0EWX	1510	822	29.68	273	44.23	106.78	0.429	6.62	5/out-in	PM
*AhZIP7.6*	arahy.QZI7QE	1207	843	30.33	280	46.91	104.11	0.399	6.62	5/out-in	PM
*AhZIP7.7*	arahy.0E1GBK	1397	822	29.88	273	45.75	104.62	0.408	6.1	5/out-in	PM
*AhZIP7.8*	arahy.NIU36G	3869	480	17.69	159	46.83	99.25	0.432	6.57	4/out-out	EMS
*AhZIP11.1*	arahy.HSP4SF	3404	1038	36.62	345	30.58	112.87	0.767	5.73	8/out-out	PM
*AhZIP11.2*	arahy.W56MR2	2492	1047	36.94	348	32.71	113.05	0.749	5.49	8/out-out	PM

^a^ Molecular weight, ^b^ amino acid number, ^c^ grand average of hydropathicity, ^d^ isoelectric points, ^e^ transmembrane domain, ^f^ plasma membrane, ^g^ endomembrane system.

**Table 2:** *Ka*/*Ks* analysis of all gene duplication pairs for *AhZIP* genes.

Gene Pairs	Duplicate Type	*Ka* ^a^	*Ks* ^b^	*Ka*/*Ks* ^c^	Positive Selection
*AhZIP6.1/6.4*	Segmental	0.1912	1.3731	0.1393	No
*AhZIP6.1/6.3*	Segmental	0.1884	1.3068	0.1442	No
*AhZIP6.2/6.4*	Segmental	0.1887	1.3548	0.1393	No
*AhZIP6.3/6.2*	Segmental	0.1859	1.2902	0.1441	No
*AhIRT1.2/1.4*	Whole-genome	0.0463	0.0956	0.4843	No
*AhZIP1.1/1.2*	Whole-genome	0.0338	0.0667	0.5068	No
*AhZIP2.1/2.2*	Whole-genome	0.0112	0.0373	0.3006	No
*AhZIP3.1/3.2*	Whole-genome	0.0393	0.0474	0.8291	No
*AhZIP3.3/3.4*	Whole-genome	0.0208	0.0861	0.2415	No
*AhZIP4.1/4.2*	Whole-genome	0.0024	0.0460	0.0525	No
*AhZIP6.3/6.4*	Whole-genome	0.0026	0.0485	0.0532	No
*AhZIP6.1/6.2*	Whole-genome	0.0013	0.0477	0.0277	No
*AhZIP7.3/7.4*	Whole-genome	0.0180	0.0848	0.2119	No
*AhZIP7.1/7.7*	Whole-genome	0.0227	0.0835	0.2717	No
*AhZIP7.5/7.6*	Whole-genome	0.0113	0.0698	0.1620	No
*AhZIP11.1/11.2*	Whole-genome	0.0077	0.0282	0.2733	No

^a^ The number of nonsynonymous substitutions per nonsynonymous site, ^b^ the number of synonymous substitutions per synonymous site, ^c^ *Ka*/*Ks* ratios.

**Table 3:** Stepwise linear regression analysis (β value) of metal accumulation and the expression of *AhZIP* genes in the roots of Fenghua 1 and Silihong (*n* = 24).

Gene Expression ^a^	[Fe]_root_ ^b^	[Fe]_shoot_ ^c^	Total Fe in Plants	% of Fe in Shoots	[Zn]_root_ ^d^	[Zn]_shoot_ ^e^	Total Zn in Plants	[Mn]_shoot_ ^f^	Total Mn in Plants	% of Mn in Shoots
*AhIRT1.1*	−0.07	−0.08	−0.52 **	0.65 **	0.21	0.60 **	0.16	1.19 ***	0.05	0.95 ***
*AhIRT1.2*	−0.39 *	0.02	0.10	0.16	0.30	0.32	0.28	0.06	0.44 *	−0.34 *
*AhZIP1.1*	0.10	0.11	−0.14	−0.07	−0.21	−0.17	−0.20	−0.06	−0.27	0.15
*AhZIP1.2*	−0.01	0.12	−0.24	−0.05	0.13	0.03	−0.12	−0.05	−0.26	0.10
*AhZIP2.1*	0.01	0.21	−0.30	0.07	0.18	0.16	−0.12	−0.04	−0.11	0.01
*AhZIP3.5*	0.06	0.27	−0.14	0.09	0.26	0.28	0.09	−0.12	−0.05	−0.14
*AhZIP3.6*	−0.12	−0.15	−0.32	0.11	0.04	0.10	−0.26	0.57 **	−0.25	0.70 ***
*AhZIP4.1*	0.00	0.13	−0.35	−0.11	−0.06	−0.02	−0.17	−0.18	−0.22	0.05
*AhZIP6.1*	−0.60 **	−0.42 *	0.02	0.06	0.02	−0.04	0.10	0.13	0.37	−0.15
*AhZIP7.2*	0.10	−0.04	0.15	0.09	0.49 *	0.06	0.45 *	0.07	0.28	−0.10
*AhZIP7.8*	0.01	0.13	−0.27	−0.08	0.21	0.13	−0.10	−0.03	−0.18	−0.01
*AhZIP11.1*	0.11	0.23	0.19	0.26	−0.05	0.43	−0.03	0.56 *	0.20	−0.14

^a^ Gene expression is calculated as −ΔΔCT, ^b^ Fe concentration in roots, ^c^ Fe concentration in shoots, ^d^ Zn concentration in roots, ^e^ Zn concentration in shoots, ^f^ Zn concentration in shoots, * *p* < 0.05, ** *p* < 0.01, *** *p* < 0.001.

## Data Availability

Not applicable.

## Supplementary Materials

The following supporting information can be downloaded at https://www.mdpi.com/article/10.3390/plants11060786/s1, Figure S1: Predicted 3D structure of peanut AhZIP proteins using the SwissModel. Models were visualized in rainbow color from N to C termini; Table S1: Analysis of the ten conserved motifs of AhZIP proteins in peanut; Table S2: The best templates of peanut AhZIP proteins selected from the SwissModel template library for building 3D structure models; Table S3: Expression profiles (TPM) of *AhZIP* genes in different tissues of peanut; Table S4: Primers of peanut *AhZIP* genes selected for RT-qPCR analysis.
