# Supplementary material for: Genome-Wide Identification and Expression Profile Reveal Potential Roles of Peanut ZIP Family Genes in Zinc/Iron-Deficiency Tolerance
Source: Plants (Basel). 2022 Mar 16;11(6):786. doi: 10.3390/plants11060786 (PMC8950646; doi:10.3390/plants11060786)
Supplement: Supplementary file 1 [file plants-11-00786-s001.zip › plants-1610898-supplementary/plants-1610898-figure.pdf]

plants

MDPI

Supplementary Material

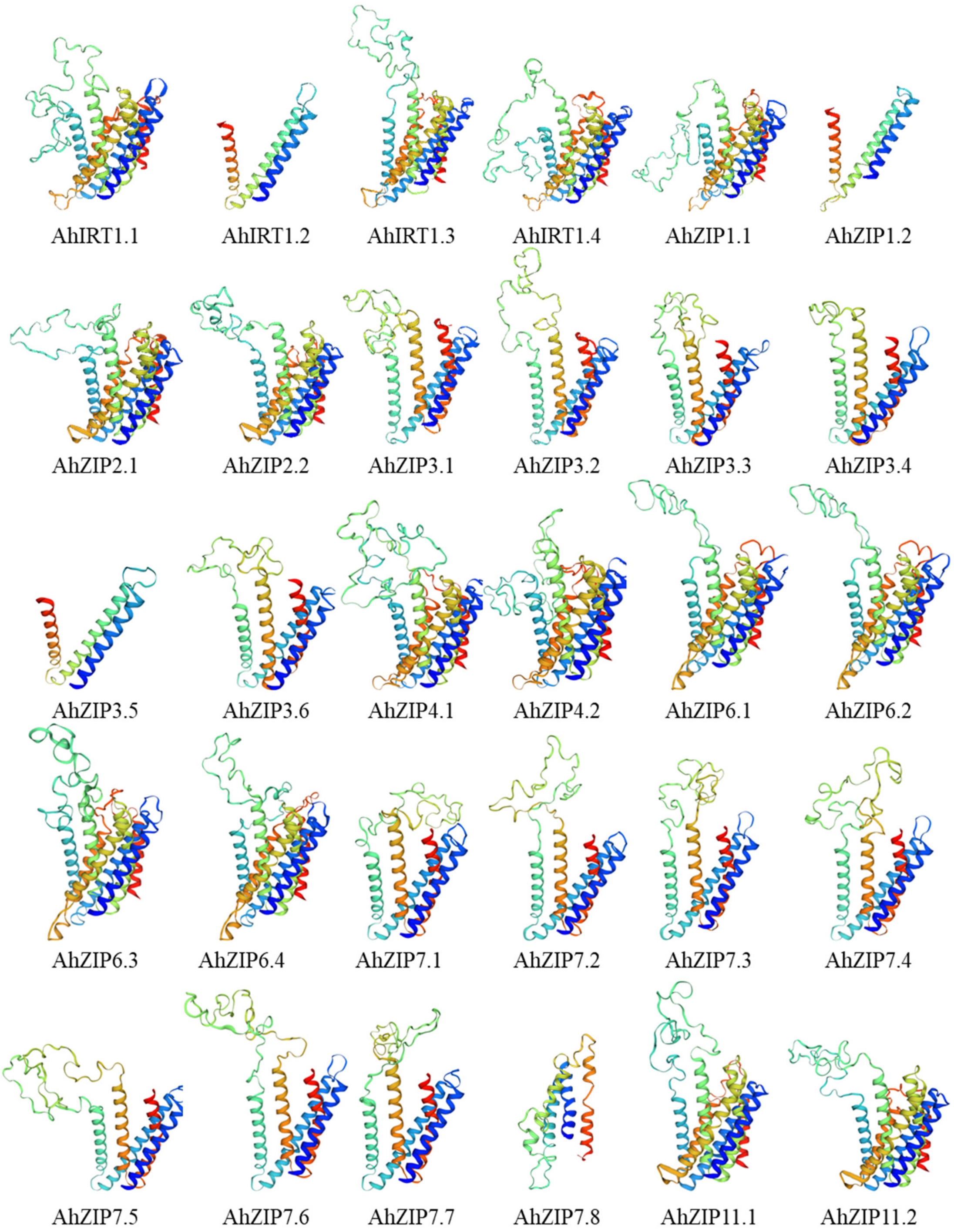

Figure S1. Predicted 3D structure of peanut AhZIP proteins using the SwissModel. Models were visualized in rainbow color from N to C termini.
